# Proton-transfer-reaction mass spectrometry (PTR-MS) for online monitoring of glucose depletion and cell concentrations in HEK 293 gene therapy processes

**DOI:** 10.1007/s10529-021-03205-y

**Published:** 2021-11-12

**Authors:** Benjamin Bayer, Andreas Maccani, Johanna Jahn, Mark Duerkop, Ewald Kapeller, Robert Pletzenauer, Barbara Kraus, Gerald Striedner, Juan A. Hernandez Bort

**Affiliations:** 1 Novasign GmbH, Vienna, Austria; 2 University of Natural Resources and Life Sciences, Vienna, Austria; 3 Gene Therapy Process Development, Baxalta Innovations GmbH, a Part of Takeda Companies, Uferstraße 15, 2304 Orth an der Donau, Austria

**Keywords:** HEK 293 cell culture monitoring, Glucose online monitoring, Cell culture PAT, Biomass soft sensor, rAAV process, PTR-MS monitoring

## Abstract

**Objectives:**

The applicability of proton-transfer-reaction mass spectrometry (PTR-MS) as a versatile online monitoring tool to increase consistency and robustness for recombinant adeno-associated virus (rAAV) producing HEK 293 bioprocesses was evaluated. We present a structured workflow to extract process relevant information from PTR-MS data.

**Results:**

Reproducibility of volatile organic compound (VOC) measurements was demonstrated with spiking experiments and the process data sets used for applicability evaluation consisted of HEK 293 cell culture triplicates with and without transfection. The developed data workflow enabled the identification of six VOCs, of which two were used to develop a soft sensor providing better real-time estimates than the conventional capacitance sensor. Acetaldehyde, another VOC, provides online process information about glucose depletion that can directly be used for process control purposes.

**Conclusions:**

The potential of PTR-MS for HEK 293 cell culture monitoring has been shown. VOC data derived information can be used to develop soft sensors and to directly set up new process control strategies.

**Supplementary Information:**

The online version contains supplementary material available at 10.1007/s10529-021-03205-y.

## Introduction

Recombinant adeno-associated viruses (rAAV) are one of the most popular viral vectors for in vitro and in vivo gene delivery used to treat diseases derived from monogenic disorders (Naso et al. 2017). A number of 149 unique clinical trials were already reported by 2019 (Kuzmin et al. 2021). Many efforts and resources have been focused on scalable manufacturing processes to achieve high titers with high full to empty capsid ratios. This emerging manufacturing technology is mostly based on triple transfection in HEK 293 cells and requires extended experience to deliver a batch-to-batch consistent product quality (Hernandez Bort 2019). To generally better understand and monitor cell behavior and product formation during cultivations, spectroscopic methods became more popular within the last decade (Rathore et al. 2010; Mercier et al. 2014; Jiang et al. 2017; Wasalathanthri et al. 2020). While spectroscopic methods in combination with chemometric modeling are attractive PAT tools, overlapping spectra or lack of sensitivity to particular components render some drawbacks in their broader applicability.

An alternative process analyzer, already evaluated with microbial systems and CHO cells is proton-transfer-reaction mass spectrometry (PTR-MS) (Bunge et al. 2008; Luchner et al. 2012; Schmidberger et al. 2014), which allows for quantitative real-time measurement of volatile organic compounds (VOCs) in the bioreactor exhaust gas stream with high sensitivity and frequency. For these systems, the identified most prominent VOCs found in the off-gas comprised methanol, acetaldehyde, ethanol, methanethiol, ethanethiol, acetone, and isoprene. The advantage of PTR-MS or online mass spectroscopic techniques, in general, is the potential to directly measure the compound's absolute concentration reaching the sensor at high sensitivity and high sampling rates. Thereby, this process analyzer gives direct insights into the cellular response to specific process conditions. To date, the investigation of VOC emission by PTR-MS in HEK cells is not covered by literature, providing an interesting and promising field. This research gap concerning VOCs becomes apparent when expanding this research topic to mammalian cells in general. Only one recent study investigated VOC emission profiles of different mammalian cell lines by using gas chromatography-mass spectrometry, providing a classification of VOCs as well as discussing the impact of the utilized media (McCartney et al. 2020). Even though a head-to-head comparison of both methods is not valid, e.g., due to different basic functionalities, sensitivity levels, and operation steps, the herein reported VOC profiles demonstrate differences between the investigated cell lines and metabolic changes during cell growth. While the major parts of these compounds were classified either as a type of alkane, esters, alcohols, oximes, or others, still 19.2% of the detected VOC signals are of unknown origin, highlighting the necessity of further research in this field.

In general, extraction of valuable process information can be achieved with unsupervised learning strategies to identify process parameters, spectroscopic wavelengths, or masses driving process variations (Bayer et al. 2020a, b). In the next step, soft sensors based on those variables combined with non-parametric or hybrid modeling approaches (Bayer et al. 2020a, b) can be set up to monitor target parameters or critical quality attributes in real-time.

Here, we describe a workflow to exclude irrelevant PTR-MS masses and simultaneously identify valuable ones for rAAV producing HEK 293 cell culture processes by applying an unsupervised learning strategy combined with process knowledge. Based on the results of this approach, a soft sensor to monitor biomass concentration was developed and compared to the state-of-the-art capacitance based biomass soft sensor. Further, a PTR-MS based decision tool for glucose depletion was developed.

## Material and methods

### Experimental setup

The experimental setup for online monitoring of VOCs consisted of a bioreactor, a sampling system, and a high-sensitivity hs-PTR-MS instrument (Ionicon Analytik, Austria). To test the functionality of the instrument and the sampling line, experiments with selected VOCs were performed.

The response time from pulse to signal increase in the PTR-MS and the reproducibility of the measurements were used as evaluation criteria. The VOCs were added either into the gas stream or to the media. These results are presented in Supplementary Fig. 1 and summarized in Supplementary Table 1.

Two HEK 293 cultivation set-ups were conducted in biological triplicates. The first one was a 4-day batch culture, and the second one was a process with transfection after 4 days growth in batch mode and subsequent cultivation for another 5 days.

### Cell line and cultivation

HEK 293 cells adapted to growth in suspension and cultivated in chemically defined serum-free medium (FreeStyle™ F17, ThermoFisher, NY, USA) were used for the production of rAAV8 vectors. Batch cultivation was performed in 20 L (WV 12 L) bioreactors at +37 °C in a humidified atmosphere containing 5% carbon dioxide and with constant stirring at 190 rpm. Transient transfection of HEK 293 cells with three plasmids containing Adenovirus 5 Helper genes, Rep2Cap8, and human FVIII sequence, respectively, was carried out with Polyethylenimine (Polysciences, PA, USA). For this, the cell culture was grown to approximately 8 × 10^6^ cells/mL and diluted with one volume of fresh medium before transfection. The fermentation producing rAAV8 particles was completed five days after transfection.

Cell density and viability were determined with a Nucleocounter NC-200 (Chemometec, Denmark). In addition, an Incyte Arc permittivity sensor (Hamilton Bonaduz, Switzerland) was used for online monitoring of the viable cell density. A correlation factor of 0.63 was applied to determine the viable cell density based on the measured permittivity. The correlation factor was determined for the used HEK 293 cell line in a previous study (data not shown).

Substrates and metabolites including glucose, lactate, glutamine, and glutamate were determined with a Cedex Bio HT Analyzer (Roche Diagnostics, Germany).

### Amino acid determination

Pre-column derivatization with 6-aminoquinolyl-N-hydroxysuccinimidyl carbamate (AQC) was used to determine and quantify amino acids (Ala, Arg, Asn, Asp, Cys, Gln, Glu, Gly, His, Ile, Leu, Lys, Met, Phe, Pro, Ser, Thr, Trp, Tyr, Val) in the fermentation supernatant. The AccQ-Tag reaction was performed according to the supplier’s instructions (UPLC Amino Acid Analysis Solution; Waters, MA, USA). Fermentation samples were diluted to the amino acid concentration range of the amino acid standard with three concentrations ranging from 50 to 400 nmol/mL. The amino acid DL-Norvaline (Sigma-Aldrich, MO, USA) was added to Amino Acid Standard Solution (5061-3330; Agilent, CA, USA) and the fermentation samples as an internal standard. The use of an internal standard with the calibration standard was used to correct volumetric errors introduced during sample preparation.

### rAAV8 ELISA

A sandwich ELISA was used to determine the product titer (rAAV8 capsid concentration) in the fermentation supernatants. Capsid particles were captured by anti-AAV8 antibodies and then detected by binding to biotinylated anti-AAV8 antibodies. Streptavidin peroxidase and a peroxidase substrate are then used for measuring bound anti-AAV8 and thus the concentration of rAAV8 capsids.

### PTR-MS installation and measurement

A commercially available high sensitivity hs-PTR-MS instrument (Ionicon Analytik, Austria) was used to analyze VOCs emanating from the fermentation process. PTR-MS allows online detection and quantification of VOCs down to parts per trillion by volume (pptv) levels. An extensive description of the PTR-MS principle and technique can be found elsewhere (Hansel et al. 1995). In brief, VOCs in the sample air are ionized by proton transfer from H_3_O^+^ ions. Molecules with a higher proton affinity than water (165 kcal/mol) are ionized, whereas the common components of air (e.g. N_2_, O_2,_ and CO_2_) have a lower proton affinity and therefore do not react. The protonated molecules weigh 1 amu more than the original compounds (m + 1). A quadrupole mass filter in combination with a secondary electron multiplier allows the ion separation according to the ion mass-to-charge ratio (m/z) which is directly linked to the mass of the VOC. The sampling system for VOC transfer from the headspace of the bioreactor to the PTR-MS instrument as well as data acquisition have already been described in detail elsewhere (Luchner et al. 2012). Example PTR-MS data (.xlsx file) of both presented cultivation set-ups are provided as Supporting Information. For process investigation and soft sensor development, time alignment of the online data (Incyte Arc and PTR-MS) and the offline data was performed.

### Exploratory data analysis

Principal component analysis (PCA) was applied to reveal hidden structures, i.e., principal components (PC), in the PTR-MS data of the HEK 293 bioprocesses, discovering relevant m/z signals explaining the variance in the data (Bro 1997). This exploratory analysis of the VOC matrix was performed with MATLAB (2020a, MathWorks, USA).

### Soft sensor development

#### Model building & validation

To estimate the cell density (response variable) in real-time a soft sensor on two PTR-MS signals (m/z 33 and m/z 59) was established. Therefore, an artificial neural network applying a Bayesian regularization algorithm was chosen. To receive smoother estimations, a 1-D online moving average filter with a window size of 10 was applied to the output of the network.

To train the model, the sampled data from the three non-transfected HEK 293 cultivations was considered, i.e., 26 biomass measurements. For internal validation of the model performance, leave-one-batch-out cross-validation was performed, i.e., the initial model was built on two cultivations and the parameters were optimized by applying it to the third cultivation. Once no further improvement was observed, the model training stopped. This procedure was repeated until every cultivation was once used for model validation.

For this procedure, the number of neurons (2–10) and hidden layers (1–3) were varied to find the optimal setting to fit the experimental data. The nodes of the hidden layer used hyperbolic tangent transfer functions, while the input and output layers used linear transfer functions. A single hidden layer with eight neurons proved to deliver the best performance with respect to the normalized root mean square error (NRMSE) in Eq. 1, where *y* is the analytical value, *ŷ* is the estimated counterpart for each sampling point (*t*), $\overline{y}$ is the mean of the measured values and *N* the total number of observations.

1 $$NRMSE[\% ]\frac{{\sqrt {\frac{1}{N} \cdot \sum {({\text{y}}_{{(t)}} - {{\hat{y}}}_{{(t)}} )^{2} } } }}{{{{\bar{y}}}}} \cdot 10$$

#### Model aggregation & model testing

To assess the risk of model misprediction, aggregation of the individual models was performed, i.e., model averaging (Freedman 1981). This approach allows selecting individual models from each of the three internal cross-validations (boots). Averaging the estimations of multiple models into one gives the operator more control in model selection and represents a robust way to investigate and deal with model uncertainties. The final bootstrap‐aggregated soft sensor consisted of two individual models, derived from different boots. To access this final model performance and the model uncertainty, the NRMSE was used along with the standard deviation (SD) (Eq. 2) and the confidence interval (CI) (Eq. 3), where *ŷ*_*bootstrap*_ is the estimation of the bootstrap aggregated model, *ŷ*_*model*_ is the estimation of the respective model, *i* the index of these models (1:2), and *n* is the number of observations for each time point.

2 $$SD_{{(t)}} = \sqrt {\frac{1}{{n - 1}} \cdot \sum ({{\hat{y}}}_{{bootstrap(t)}} - {{\hat{y}}}_{{model(i)_{{(t)}} }} )^{2} }$$

3 $$CI_{{\left( t \right)}} = {{\hat{y}}}_{{bootstrap(t)}} \pm SD_{{\left( t \right)}}$$

Testing of the developed bootstrap‐aggregated soft sensor model was performed by introducing an external test set, i.e., the three transfected HEK 293 cultivations (45 biomass measurements). The complete workflow for the soft sensor development was developed with MATLAB (2020a, MathWorks, USA).

## Results and discussion

### rAAV production process

**Fig. 1:**
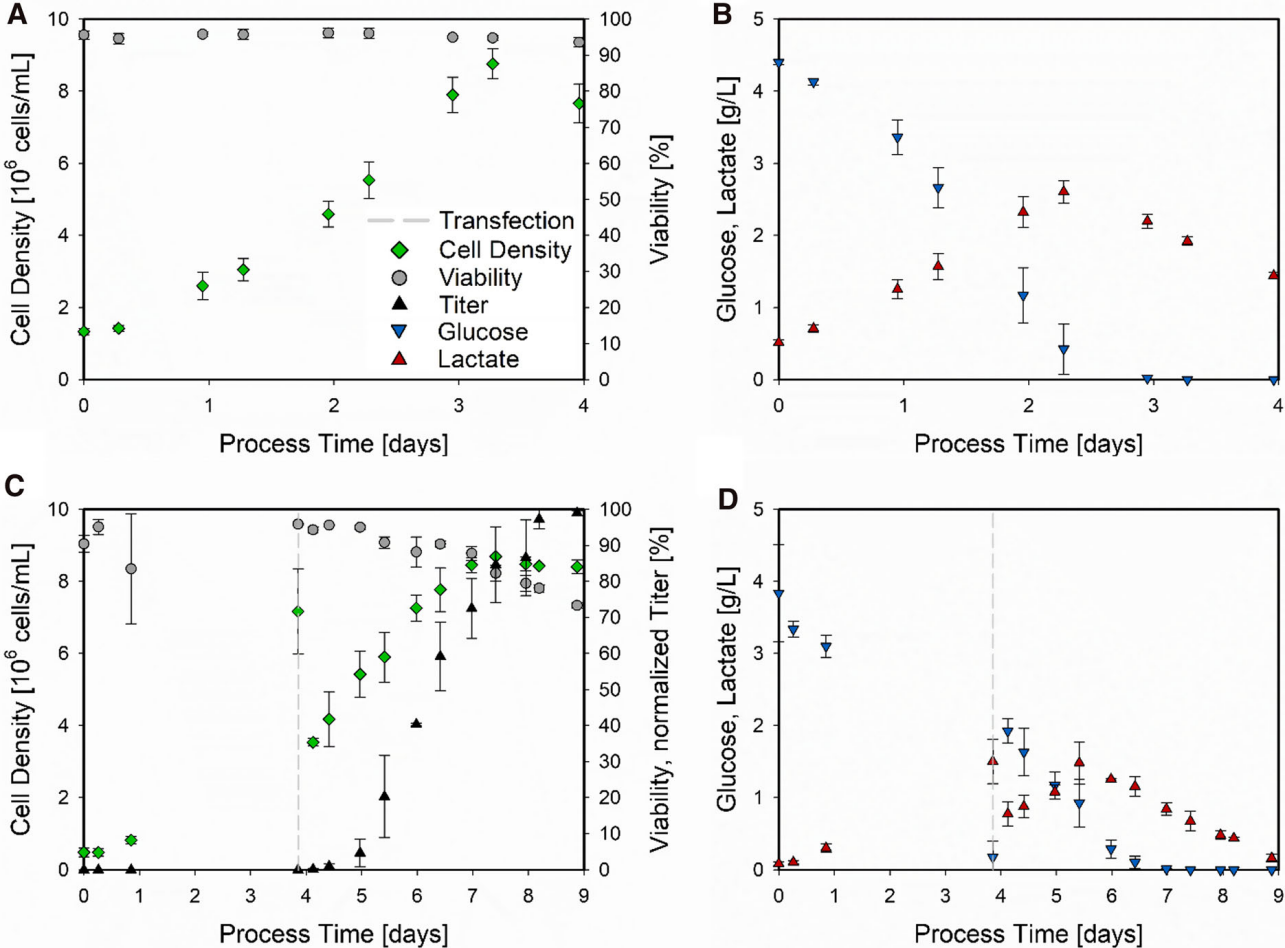
Trends of non-transfected and transfected HEK 293 bioprocesses. For the non-transfected bioprocess (N = 3), the cell density (green diamonds) and viability (grey circles) are presented as a function of the process time (**A**), as well as the glucose (blue triangles) and lactate concentration (red triangles) (**B**). For the transfected bioprocess (N = 3), the timepoint of transfection (dashed grey line) is indicated and the normalized titer (black triangles) is presented along with the cell density and the viability (**C**). Glucose and lactate concentrations are similarly displayed (**D**). The displayed values are the average of the respective triplicate ± SD

To guarantee a sound evaluation of the HEK 293 bioprocesses and estimate the biological and analytical deviation, the average values and the SD of the non-transfected and transfected bioprocess triplicates are presented. For this evaluation, the cell density, viability, glucose, and lactate concentrations, and the titer of the transfected cultivations were considered and are shown in Fig. 1.

The non-transfected triplicate (Fig. 1A,  B) displayed overall reproducible analytical results, indicated by small SDs. A similar picture emerged from the transfected triplicates (Fig. 1C, D). Even though the measured viability on the first day displayed a high SD, this can be considered as an outlier, and also the SD of the product titer is set in the range of the analytical measurement error. Overall, these presented results in Fig. 1 demonstrate high reproducibility of the bioprocesses, which is important for the further evaluation of the PTR-MS and investigation of the cellular behavior. Moreover, the herein presented cell growth and product formation are comparable to already published literature (Chahal et al. 2014; Grieger et al. 2016; Blessing et al. 2019; Zhao et al. 2020), constituting a well-performing manufacturing platform.

### VOC selection procedure

**Fig. 2:**
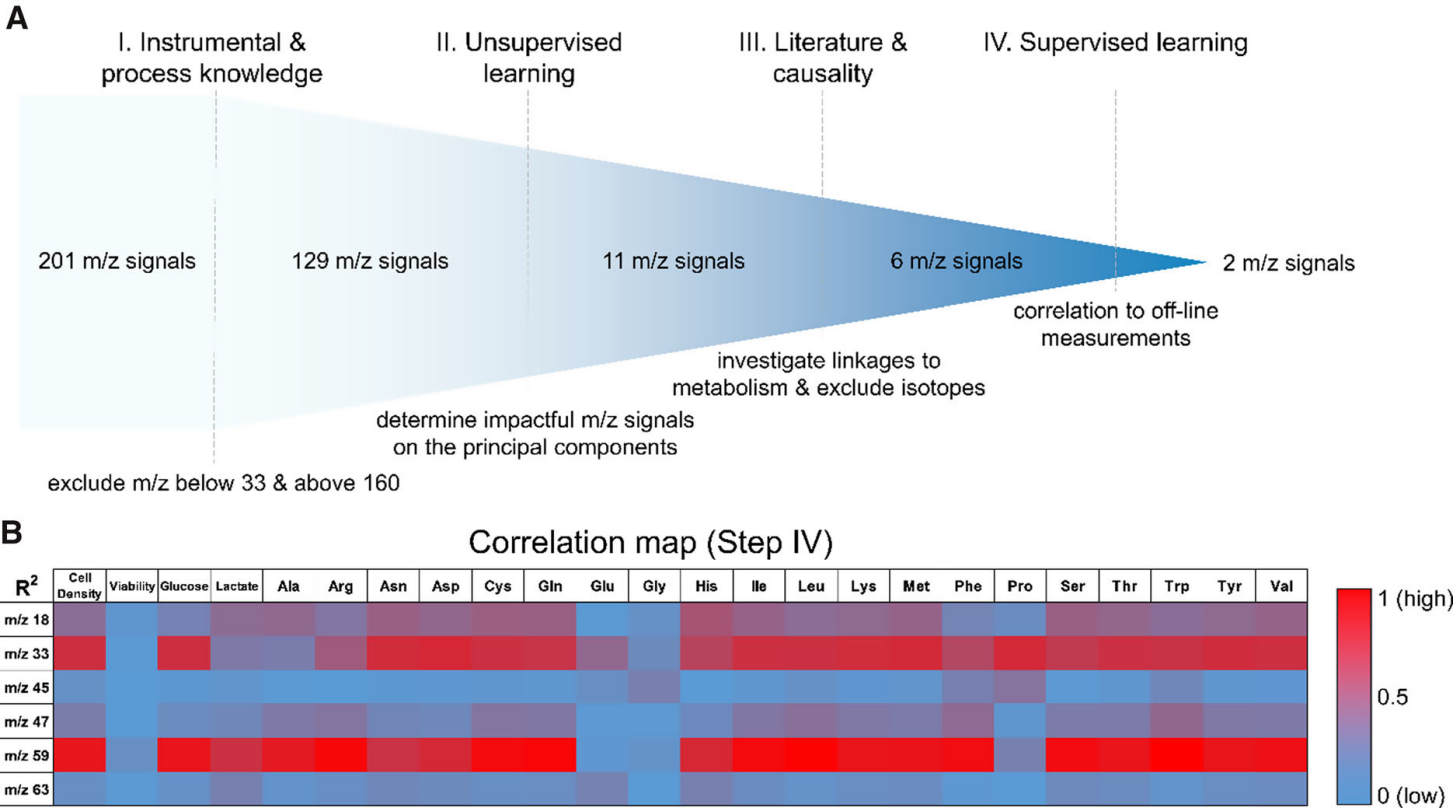
Selection procedure of meaningful PTR-MS signals out of the entire VOC matrix. The complete workflow (step I–IV) and the conducted actions, reducing the potential candidate m/z signals are given (**A**). Initial exclusion of these signals was performed with existing knowledge (step I), followed by unsupervised learning (PCA, step II). The identified impactful m/z signals were compared to known VOCs from literature and assigned according to the highest likelihood or excluded if no linkage could be found (step III). For step IV (supervised learning), the correlation between the remaining m/z signals and the analytical measurements is provided as a heatmap (**B**)

For the selection of meaningful PTR-MS signals out of the entire VOC matrix, a structured workflow was developed. This workflow consists of four individual steps with an increasing specificity to find and select valuable m/z signals from the original 201 signals, as shown in Fig. 2.

Using the structured approach from Fig. 2A, the initial 201 m/z signals of the VOC matrix were reduced to two m/z signals. Due to existing knowledge about the device and the process (step I), the VOC matrix could be reduced to 129 m/z signals by excluding irrelevant signals, i.e., signals below m/z 33 are only derived from the device itself (except m/z 18) and above m/z 160 no responses were detected by the device (ppb values around 0–4). On this reduced VOC matrix, unsupervised learning was applied (step II). Here, PCA revealed that out of the remaining signals, only 11 m/z signals are responsible for almost all the variance in the data, which were further used in the workflow. For these 11 m/z signals, literature research and causality analysis were performed (step III) to identify matching m/z values with known and reported VOCs from literature (Bunge et al. 2008; Luchner et al. 2012; Schmidberger et al. 2014) and if possible causal linkages, which allowed to reduce this number even further to 6 m/z signals. In short, the important m/zs, and the most likely corresponding VOCs according to known substances from the literature (in brackets), were found in 18 (ammonia), 33 (methanol), 45 (acetaldehyde), 47 (ethanol), 59 (acetone) and 63 (ethanethiol). More detailed information about selection step II (unsupervised PCA) and III (literature and causality analysis) are presented in Supplementary Fig. 2. The final step IV was performed to determine which of these 6 m/z signals do not only contain useful information but can also be used for supervised learning, i.e., displaying correlations to analytical measurements. The heatmap (Fig. 2B) revealed that the timely progression of two m/z signals, m/z 33 and m/z 59, displayed the highest correlations to trends of analytical measurements, e.g., the cell density, and were, therefore, suitable for soft sensor development.

While the herein presented workflow is generally applicable, the outcome is specific for the system and used media. The utilization of different systems or media might lead to the identification of other m/z signals, e.g., due to an activated pathway by different media components. However, to more closely investigate and verify these findings, and also with respect to causality, additional experiments with different systems and media should be performed in future studies.

### PTR-MS for cell density soft sensing

**Fig. 3:**
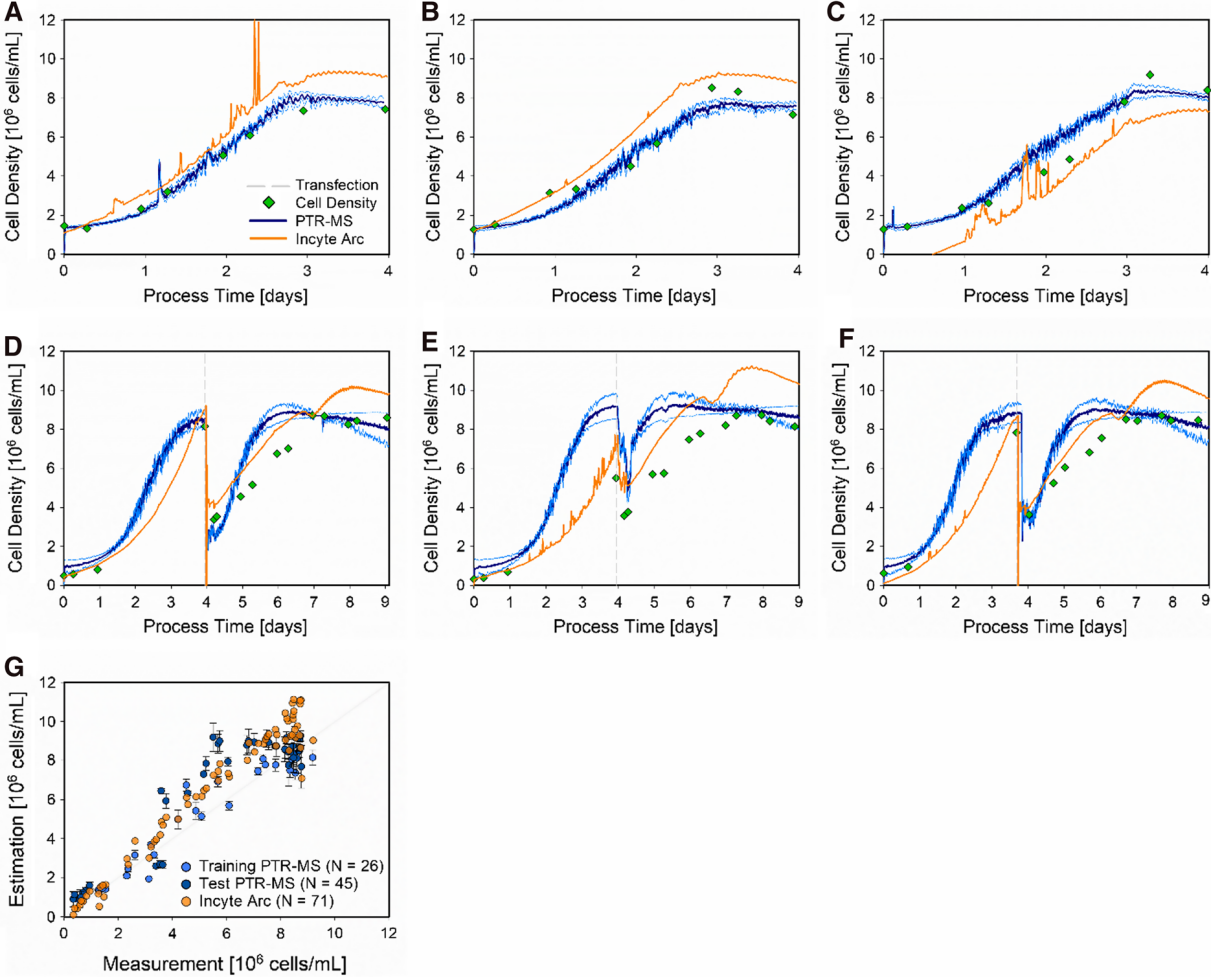
Soft sensor performance comparison for estimating the cell density in HEK 293 bioprocesses. The cell density (green diamonds) is presented for the entire process time of the three non-transfected cultivations (**A**–**C**) and the transfected cultivations (**D**–**F**). For the transfected cultivations, the timepoint of transfection (dashed grey line) is indicated. For each cultivation, the soft sensor estimation of the PTR-MS (blue lines) and the Incyte Arc probe (orange lines) are given. The scatter plot (**G**) provides an overview of the entire performance comparison, displaying the analytical values (x-axis) versus the estimated values (y-axis) for the PTR-MS training data (light blue circles), PTR-MS test data (dark blue circles), and the Incyte Arc probe (orange circles). For the time-resolved presentation of the PTR-MS soft sensor (**A–F**), the CI (light blue lines) and the error bars indicating the SD in the scatter plot are given

Based on the results from the VOC selection procedure (Fig. 2), a metabolic soft sensor based on m/z 33 and m/z 59 was developed, estimating the cell density in real-time. Since the soft sensor derived from the Incyte Arc probe is considered as standard for cell density estimations, it was utilized as the benchmark performance and directly compared. This performance comparison for the two soft sensors estimating the cell density in the HEK 293 bioprocess triplicates is presented in Fig. 3.

The comparison of the soft sensors in Fig. 3, estimating the cell density based either on PTR-MS data or the Incyte Arc probe, has shown that the PTR-MS delivers more reliable results. The triplicate, used for training the PTR-MS soft sensor (Fig. 3A–C), were accurately estimated (NRMSE 15.8%) with a low risk of model uncertainties indicated by small CIs. The test set (Fig. 3D–F) proved to be more challenging (NRMSE 24.4%), but still performed superior compared to the Incyte Arc soft sensor (NRMSE: 48.7%), which almost constantly under- or overestimated the analytically derived values (Fig. 3G).

However, the PTR-MS soft sensor displayed two shortcomings in the test set. First, the cell density in the second test cultivation at the time point of transfection (Fig. 3E) was overestimated, and second, the cell growth after the transfection until the plateau phase was reached, was generally overestimated. This demonstrates that for the implementation of a more powerful and highly robust soft sensor, a design space must be chosen and characterized. In that way, variance is introduced to the model and such altered cultivation trends can be understood and therefore accurately estimated by the model. Besides a more robust process model, these additional experiments would also increase the confidence about the potentially identified VOCs, i.e., varying trends at other process settings, affecting cellular responses, might further verify our selection or indicate misinterpreted m/z signals.

Nevertheless, the test set of the developed PTR-MS soft sensor highly differed from the training set, e.g., different starting cell densities and it also contained the time after transfection (extrapolation of the non-transfected bioprocess used for model training). This is not only leading to a bigger size of the test data set but also metabolic conditions and cellular behavior the model has never seen before, which provides a good indication for the soft sensor performance Despite these challenges, the developed PTR-MS soft sensor proved to be well-suited for online monitoring the cell density in HEK 293 bioprocesses, since the unspecific m/z measurement can immediately be interpreted by the trained algorithm into specific concentrations. This enables real-time estimation of an important process variable without the need of taking a sample and analytical time delay, e.g., as is the case by utilizing hemocytometry or thermogravimetric analysis.

### PTR-MS as a beneficial process monitoring tool and control action indicator

**Fig. 4:**
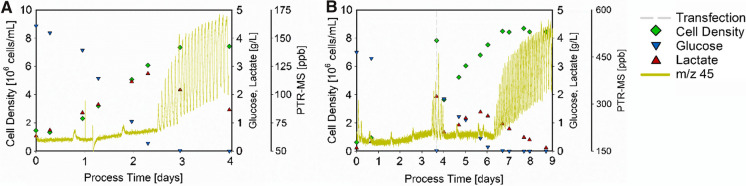
Trends of bioprocess variables along with m/z 45 for a non-transfected and transfected HEK 293 cultivation. The cell density (green diamonds), glucose (blue triangles), and lactate concentrations (red triangles) are displayed along with m/z 45 (dark yellow lines) for a non-transfected (**A**) and a transfected HEK 293 cultivation (**B**). For the transfected cultivation, the time point of transfection (dashed grey line) is indicated

The four m/z signals, which are of importance according to the unsupervised PCA (Fig. 2A, step II) but did not show correlations to offline measurements were investigated for other potential applications. The graphical comparison between the trend and behavior of these four VOCs (m/z 18, m/z 45, m/z 47, and m/z 63) throughout a non-transfected cultivation is presented in Supplementary Fig. 3. In this process, m/z 45 (potentially acetaldehyde) proved to be the most suitable VOC for process monitoring and as a control action indicator. To demonstrate the application possibilities, the trend of m/z 45 along with the cell density, glucose, and lactate concentration is presented for an exemplary non-transfected and transfected HEK 293 cultivation in Fig. 4.

The measured concentrations of m/z 45 were almost noise-free and steady until the glucose in the cultivation was about to be depleted, for which the exact time point can only be assumed due to the resolution of the sampling interval. Close to glucose depletion, the signal sharply started to fluctuate, displaying increasing values by 185% compared to the steady trend (from ~ 60 to ~ 170 ppb) for the non-transfected cultivation (Fig. 4A). This behaviour was observed after approximately 2.5 days, where cell densities reached a plateau in all the observed cultivations. However, since it is almost impossible to exactly determine the cultivation timepoint at which glucose was depleted by offline measurements, the finding of m/z 45, as a simple online process monitoring tool for glucose depletion, is of high value. Additionally, with this reliable online indicator, the optimal time for the transfection can be precisely determined in real-time and is not depending on the sampling procedure. In addition, due to this prompt online available information about glucose depletion and as a result, the possibility to rapidly react, the cells will not go into starvation for longer periods, thus fewer process variability after transfection, and therefore more robust and consistent bioprocesses can be expected.

The same behavior was observed in the transfected cultivation (Fig. 4B). Herein, the glucose was depleted after ~ 4 days and m/z 45 also displayed fluctuating values, sharply increasing by 185% compared to the steady trend (from ~ 170 to ~ 485 ppb). Moreover, after transfection and the hereby newly added glucose, the fluctuations quickly stopped and the trend from the non-transfected phase before was restored, indicating that the cell metabolism was able to recover from glucose starvation. Interestingly, the same fluctuations started again once the glucose was depleted in the production phase (shortly after day 6), rising by roughly 220% (from ~ 170 to ~ 550 ppb). These first findings indicate that falling below a critical glucose level triggers m/z 45 to be emitted in waves, which can be a hint to a drastically altered cellular ‘breathing’ pattern, e.g., due to unfavored process conditions and enhanced stress levels. A further factor to be considered for this behavior is the onset of lactate consumption as an alternative fuel, which potentially leads to these fluctuations. To gain certainty about this reasoning, further cultivations, which are carried out and monitored beyond the complete depletion of glucose and lactate (e.g., for some additional days) could provide more information and evidence about the origin of m/z 45 and its emission profile with even higher levels of stress.

**Fig. 5:**
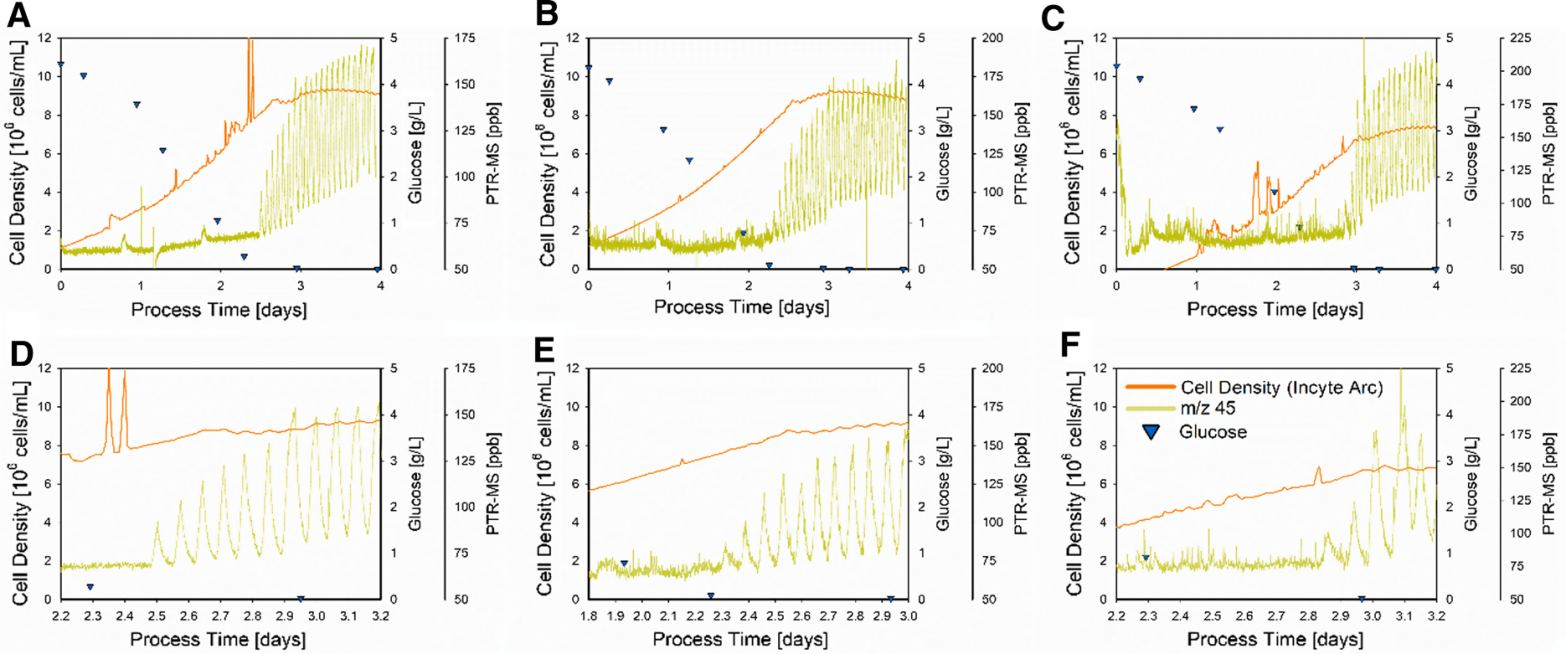
Comparison of two online indicators for glucose depletion in HEK 293 cultivations. The glucose concentration (blue triangles), the PTR-MS signal for m/z 45 (dark yellow lines), and the cell density estimation of the Incyte Arc probe (orange lines) are given for each non-transfected HEK 293 cultivation (**A**–**C**). The time window of interest for each cultivation is displayed beneath the overall presentation of the respective cultivation (**D**–**F**)

The PTR-MS signal of m/z 45 has been demonstrated to be well-suited for rapidly indicating glucose depletion in Fig. 4, i.e., no further cell growth and therefore the optimal time of transfection. The resolution of the offline measurement was not high enough to precisely determine this time point. Therefore, the online determination of this time point in the process as precisely as possible is of high value, especially concerning process consistency. Since the Incyte Arc probe may also be suitable to indicate glucose depletion, i.e., displaying a plateau in the cell density, it was considered as a further reference and compared. This comparison of both online signals along with the glucose concentration for the non-transfected HEK 293 bioprocess triplicate is shown in Fig. 5 for the whole process time and also for the relevant time windows.

As it can be seen in Fig. 5, precise capture the time of glucose depletion is a tremendous challenge with respect to sampling and the analytical time delay. Since more than two samplings per day are rarely meaningful in mammalian processes, the high benefit of such an online indicator is highlighted again. The expected responses of both online sensors to glucose depletion are measurable for the three different cultivations (Fig. 5A–C). To gain deeper insights, the narrowed time window of each cultivation was investigated and the response time of the two sensors was compared. The time window was chosen concerning the availability of offline measurements, guaranteeing a sound statement (1 to 1.2 days).

For the first cultivation (Fig. 5A , D), the PTR-MS displayed the first peak of m/z 45 after 2.5 days, indicating glucose depletion. The Incyte Arc probe displayed a small drop in the estimation of the cell density after 2.75 days, which rose again until a plateau was reached around day 3. Similar observations were made for the second cultivation (Fig. 5B, E) at which the first peak of m/z 45 was measured after 2.3 days. For the Incyte Arc probe, the same drop as before was observed after 2.6 days until the plateau was reached at day 3. The same behavior was observed in the third cultivation (Fig. 5C, F). Here, the first peak of the PTR-MS was measured after 2.85 days, while the drop in the Incyte Arc signal appeared after 3.1 days and the plateau phase after 3.5 days.

The direct comparison of the two probes showed that the PTR-MS, more precisely m/z 45, clearly responds faster than the Incyte Arc probe with respect to the response time indicating glucose depletion. The first response in the Incyte Arc signal was detectable 0.25–0.3 days after the first measurable peak of the PTR-MS. After this initial drop in the Incyte Arc signal, it took 0.25–0.4 additional days until a plateau was reached, i.e., after the first observed peak in the PTR-MS, 0.5–0.7 additional days elapsed until the Incyte Arc probe indicated the plateau phase. Moreover, besides the assumed almost immediate response after glucose depletion, the peaks of m/z 45 were way more explicit compared to the trends provided by the Incyte Arc probe.

Both these characteristics, the significantly faster response time and the explicit nature of the peaks, emphasize the use of the PTR-MS as a rapid and well-suited online indicator for glucose depletion and therefore to get the perfect transfection time avoiding that cells go into starvation. However, as already discussed above, the origin of m/z 45 close to glucose depletion needs to be uncovered in future studies to verify this signal as a generic and robust process indicator, i.e., the mechanism of triggering this stress response needs to be investigated.

**Fig. 6:**
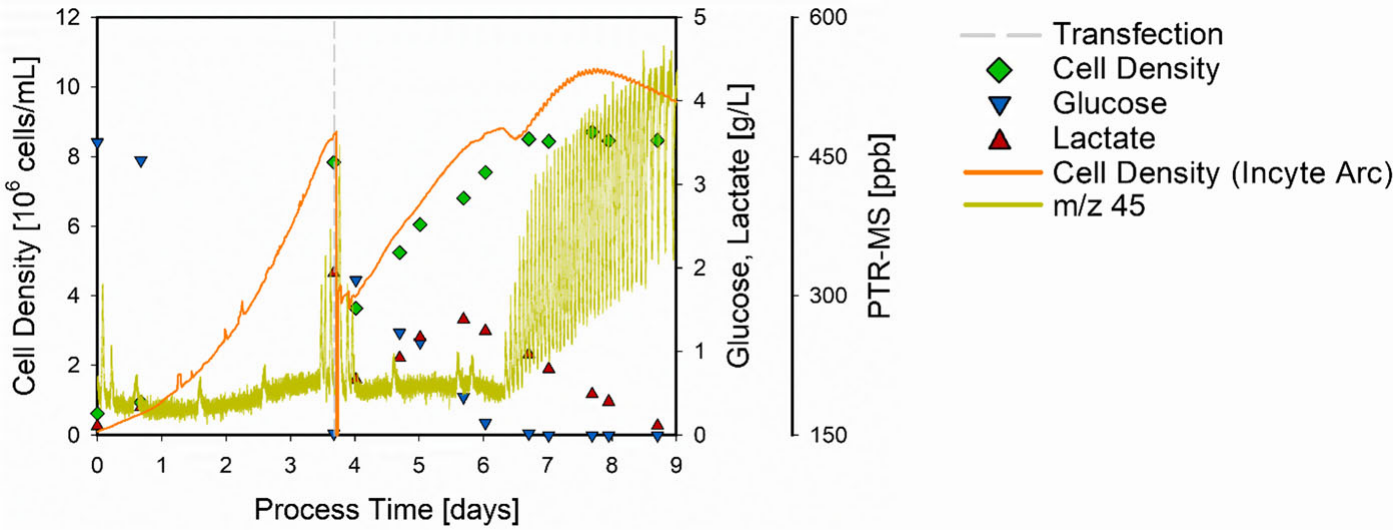
Holistic presentation of a transfected HEK 293 cultivation including key process variables. The cell density (green diamonds), glucose (blue triangles), and lactate concentrations (red triangles) are given as a function of the process time along with the PTR-MS signal for m/z 45 (dark yellow line) and the cell density estimation of the Incyte Arc probe (orange line). The time point of transfection (dashed grey line) is indicated

Since Fig. 4B showed that the steady trend of m/z 45 can be recovered once glucose is again added to the cultivation, also the usability of this online indicator for glucose depletion for the transfection phase was investigated. A complete transfected HEK 293 cultivation including the key process variables along with the online signals of m/z 45 and the Incyte Arc probe is shown in Fig. 6.

Considering the presentation of an entire transfected HEK 293 cultivation in Fig. 6, all observations and findings from the previous results were verified and retrieved. At the time point of transfection (day 3.7) glucose was already depleted, which was indicated by the PTR-MS 0.2 days in advance (at day 3.5) but not recognized by the Incyte Arc probe. Moreover, the fluctuating PTR-MS signal recovered after glucose was again added to the cell culture, restoring the previous level until glucose was again depleted from the medium (offline measurement at day 6.7). The first peak of m/z 45 was already detected on day 6.35 and the drop in the Incyte Arc signal at day 6.5 was also observed. Moreover, the Incyte Arc probe estimated an increasing cell density until a plateau was reached at day 7.75, even though glucose was depleted more than a day before, and additionally, a steady cell density was reached.

This again highlights the faster response time (assumingly in real-time) and the higher sensitivity of the PTR-MS to indicate glucose depletion and thereto related cellular restrictions. Since the steady trend of the m/z 45 signal can be recovered by adding glucose to the cultivation, this effect cannot only be used as an online indicator for the optimal transfection time but moreover during the transfection phase. For instance, potentially as a closed-loop feedback control to add glucose on demand, improving the cellular conditions and receiving higher yields.

## Conclusion

The presented structured workflow facilitates the identification of valuable m/z signals from the entire VOC matrix and highlights the added value of PTR-MS as a versatile online monitoring tool in rAAV HEK 293 gene therapy bioprocesses. The herewith developed PTR-MS based soft sensor enables real-time monitoring of the cell density with a smaller NRMSE (24.4%) than the benchmark sensor (NRMSE 48.7%). Compared to conventional offline analytics, this timely advantage also provides the opportunity to act as promptly as possible in case time-critical actions are required, while simultaneously eliminating the contamination risk due to sampling procedure. Additionally, m/z 45 was identified to be a highly sensitive and rapidly responding online indicator for glucose depletion. This very simple and straightforward online indicator can be used to determine the optimal time point of transfection and potentially enable a closed-loop feedback control to add glucose on demand, avoiding cell starvation and improving process performance.

## Supplementary Information

Below is the link to the electronic supplementary material.

Supplementary file1 (XLSX 25040 kb)

Supplementary file2 (DOCX 5428 kb)

## Abbreviations

AQC: 6-Aminoquinolyl-*N*-hydroxysuccinimidyl carbamate
CI: Confidence interval
m/z: Ion mass-to-charge ratio
NRMSE: Normalized root mean square error
PC: Principal component
PCA: Principal component analysis
pptv: Parts per trillion by volume
PTR-MS: Proton-transfer-reaction mass spectrometry
rAAV: Recombinant adeno-associated virus
SD: Standard deviation
VOC: Volatile organic compound
