# Supplementary material for: Proton-transfer-reaction mass spectrometry (PTR-MS) for online monitoring of glucose depletion and cell concentrations in HEK 293 gene therapy processes
Source: Biotechnol Lett. 2021 Nov 12;44(1):77–88. doi: 10.1007/s10529-021-03205-y (PMC8854141; doi:10.1007/s10529-021-03205-y)
Supplement: Supplementary file 2 — Supplementary file2 (DOCX 5428 kb) [file 10529_2021_3205_MOESM2_ESM.docx]

**Supporting Information**

**VOC spiking experiments – PTR-MS and sampling line functionality test**

The PTR-MS device is introduced in the Materials & Methods section of the main manuscript. Since reproducibility of measurements and appropriate response times to changes in the measured m/z signals are important, spiking experiments with three VOCs were performed (acetaldehyde (m/z 45), ethanol (m/z 47), and acetone (m/z 59) respectively). These experiments were performed to demonstrate that the response time from VOC addition to signal rise in PTR-MS is appropriate for the physiological behavior of HEK 293 cells in the culturing process. In addition, replicate experiments allowed for an evaluation of the reproducibility of the measurements. For the spiking experiments into the gas phase, the pure VOC solutions were used, except for acetaldehyde, for which a 1:5 dilution was used due to its high volatility. The concentrations of the VOC solutions used for the spiking experiments into the liquid phase were 0.12 % for acetaldehyde, 0.24 % for ethanol, and 0.024 % for acetone. These VOCs were injected into the gas stream (2 ml of the aspirated headspace of the respective VOC, collected by using a syringe) and into the media (5 ml of the respective solution).

Each spiking experiment was repeated at least three times for meaningful evaluation and is displayed in Fig. S1. Herein, the PTR-MS measurements are normalized (zero to one) to enable simpler comparison.

**
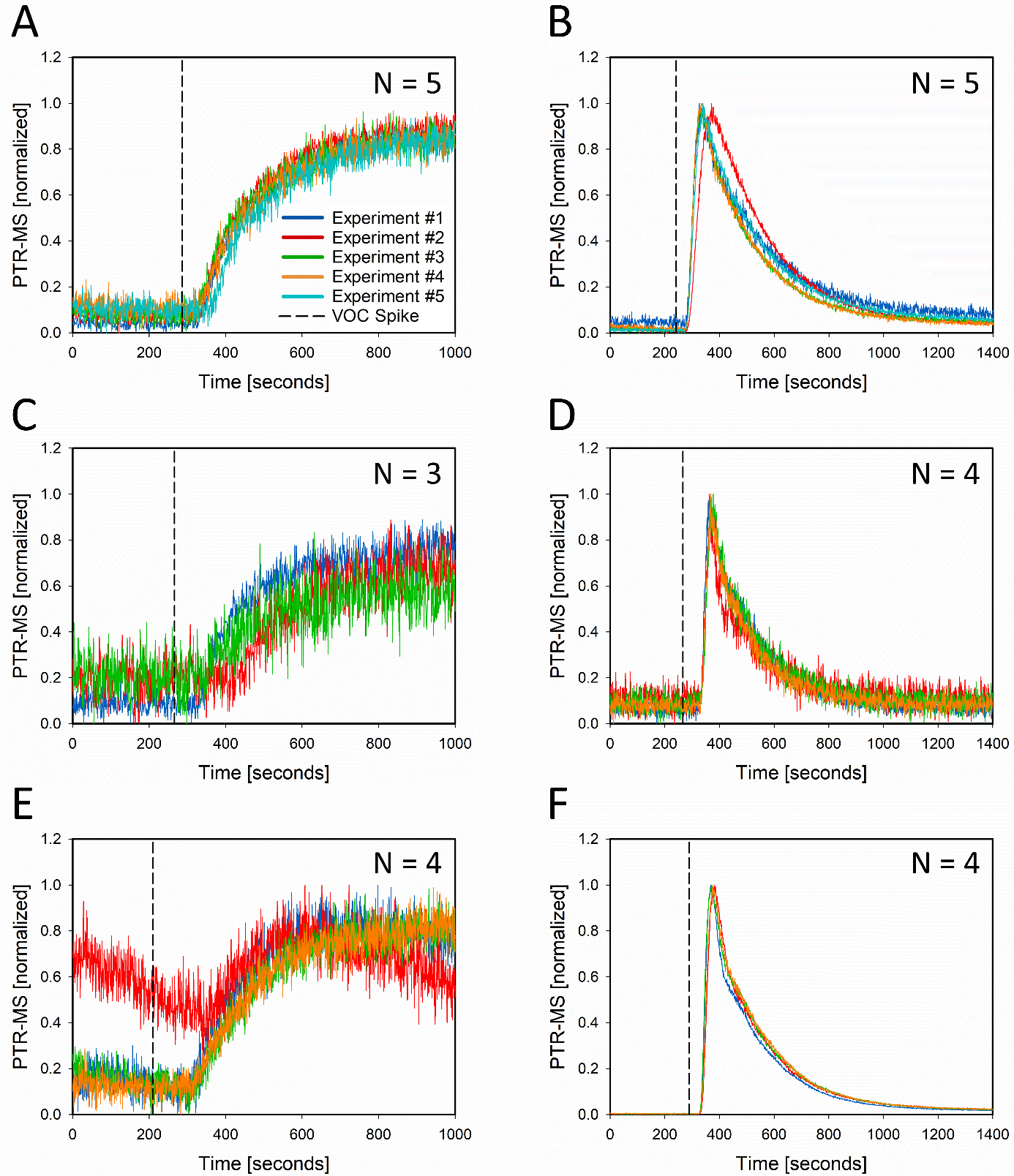
Fig. S1.** Characterization of the PTR-MS response time by VOC spiking experiments. Three VOCs were injected into the gas stream and the media to investigate the response time and outgassing characteristics: acetaldehyde (m/z 45, A: media; B: gas), ethanol (m/z 47, C: media; D: gas), and acetone (m/z 59, E: media; F: gas).

As displayed in Fig. S1, the injections directly into the media resulted in different and specific response times for each VOC, i.e., acetaldehyde was detected after 58 (± 9) seconds (Fig. S1A), ethanol after 101 (± 19) seconds (Fig. S1C) and acetone after 108 (± 15) seconds (Fig. S1E). Contrary, highly similar results were obtained for the injections into the gas stream. Herein, the PTR-MS was on average able to detect acetaldehyde (Fig. S1B) in 43 (± 2) seconds, ethanol (Fig. S1D) in 55 (± 5) seconds, and acetone (Fig. S1F) in 41 (± 1) seconds. These results are summarized in Table S1.

**Table S1.**  Response time of the PTR-MS for different spiked compounds and phases. The indicated response time presents the mean value from all replicates ± SD

| Compound | Reactor Phase | Response Time [s] |
| --- | --- | --- |
| Acetaldehyde | Liquid (N = 5) | 58 ± 9 |
|  | Gas (N = 5) | 43 ± 2 |
| Ethanol | Liquid (N = 3) | 101 ± 19 |
|  | Gas (N = 4) | 55 ± 5 |
| Acetone | Liquid (N = 4) | 108 ± 15 |
|  | Gas (N = 4) | 41 ± 1 |

The presented response times for the spiked VOCs along with the standard deviation of the replicates indicate high reproducibility, which is the prerequisite for implementing such a process analyzer in cell culture processes. Additionally, changes in the metabolism of HEK 293 cells appear slower than the presented response times in Table S1. Based on these results it can be concluded that the PTR-MS is suitable to monitor and detect changes in the VOC matrix of HEK 293 bioprocesses with sufficient sensitivity and response time.

**Unsupervised learning using the PTR-MS VOC matrix**

The selection procedure of suitable VOCs for soft sensing and advanced online monitoring is introduced in the Results & Discussion section of the main manuscript (Fig. 2). The more detailed information about the unsupervised learning (Fig. 2A, step II) is provided in Fig. 2S, i.e., PCA results of the VOC matrix. For the PCA of the already reduced VOC matrix (129 m/z signals), different data partitions were considered as inputs to detect potential deviations in the explained variance and the respective loadings:

- All non-transfected cultivations (N = 3)
- All transfected cultivations (N = 3)
- All cultivations (N = 6)

**
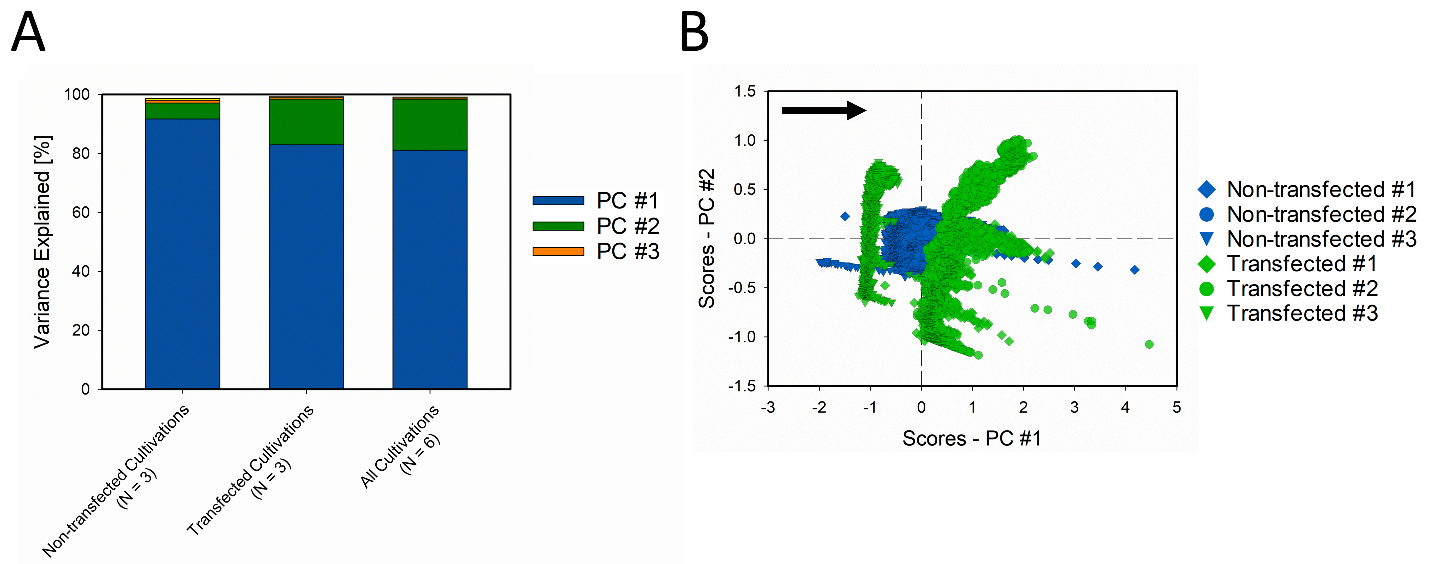
Fig. S2**. PCA results of the VOC matrix from HEK 293 bioprocesses. The stacked explained variance of the first three PCs is displayed in blue, green, and orange, respectively (A). Results are shown for all non‑transfected cultivations (left bar), all transfected cultivations (middle bar), and all cultivations (right bar). The scores for the first two PCs of the non-transfected (blue symbols) and transfected bioprocess triplicates (green symbols) are presented as scatter plot (B) along with the direction (black arrow).

In all cases, two components were sufficient for explaining more than 97 % of the total variance in the data (Fig. S2A). The different input data sets to the PCA did only reveal minor changes in the distribution of the explained variance between the first and second PC. The first PC of the data set containing the non‑transfected cultivations was already responsible for 91.7 % of the explained variance, while the second (5.4 %) and third PC (0.9 %) did only account for a minor part. The data set containing the transfected cultivations displayed a higher variance in the VOC matrix and therefore also a slightly different distribution for the explained variance by the PCs. The first PC was accountable for 82.9 %, the second for 15.5 %, and the third for 0.4 % explained variance. The third data set, containing all cultivations, displayed similar distributions as obtained with the second data set, i.e., the first PC is accountable for 81.1 %, the second for 17.3 %, and the third for 0.4 % explained variance in the data. This increased variance in the data of the transfected cultivations, especially PC #2, is also visible in the scatter plot of the first two PCs (Fig. S2B). While the scores of the non-transfected triplicate were all close by, the scores of the transfected triplicate displayed a similar but wider spread, as already indicated in Fig. S2A.

In addition, the respective m/z signals can be identified via their loadings for further utilization and irrelevant m/z signals are excluded from subsequent steps. According to PCA, the same eleven m/z signals were responsible for the loadings regardless of the input VOC matrix and were therefore chosen for literature and causality analysis in Fig. 2A (step III). This next selection step revealed that even though eleven m/z signals were found to be important in the PCA, only six were chosen to further proceed with after the literature and causality analysis. The following m/z signals were matched with known VOCs from the literature and assigned according to their highest likelihood:

- m/z 18: Ammonia
- m/z 33: Methanol
- m/z 45: Acetaldehyde
- m/z 47: Ethanol
- m/z 59: Acetone
- m/z 63: Ethanethiol

The following m/z signals were excluded:

- m/z 37: H_2_O cluster
- m/z 38: not assignable, a steady trend with low ppb values (~5)
- m/z 43: not assignable, low ppb values (0 - 68)
- m/z 57: not assignable, a steady trend with low ppb values (~12)
- m/z 60: isotope of m/z 59

Concerning these remaining six m/z signals and a linkage to cell metabolism, open discussion points still should be investigated and resolved in future studies. For example, the presence of ethanol (m/z 47) in the off-gas can be traced back to process operations and not to the cells, while the detection of ethanethiol, is highly unlikely even at very low ppb amounts as observed herein. Moreover, also the presence of methanol (m/z 33) in mammalian bioprocesses is from a metabolic point of view not obvious. A similar picture is derived for the presence of acetaldehyde (m/z 45), which is reasonable for microbial fermentations but can be critically questioned for mammalian bioprocesses. These m/z signals, which may not directly be connected to cell metabolism could origin from interactions with media components. The herein discussed observations can also be investigated in the provided exemplary PTR-MS cultivation data. However, to resolve and shed light on these mentioned issues, additional experiments with different media compositions in future studies may prove to be highly useful and provide further evidence.

**Selection of a metabolic marker**

The identified and chosen metabolic marker (m/z 45, most likely acetaldehyde) for monitoring glucose depletion is displayed in the Results & Discussion section of the main manuscript (Fig. 4). To highlight and validate this choice, the complete comparison between all remaining four m/z candidates not chosen for supervised learning (Fig. 2, step IV) is displayed for one exemplary non-transfected cultivation in Fig. S3. To provide enough details about the metabolic state of the HEK 293 cells, guaranteeing a sound decision, the cell density, glucose, and lactate concentrations are displayed along with the respective m/z signal.

**
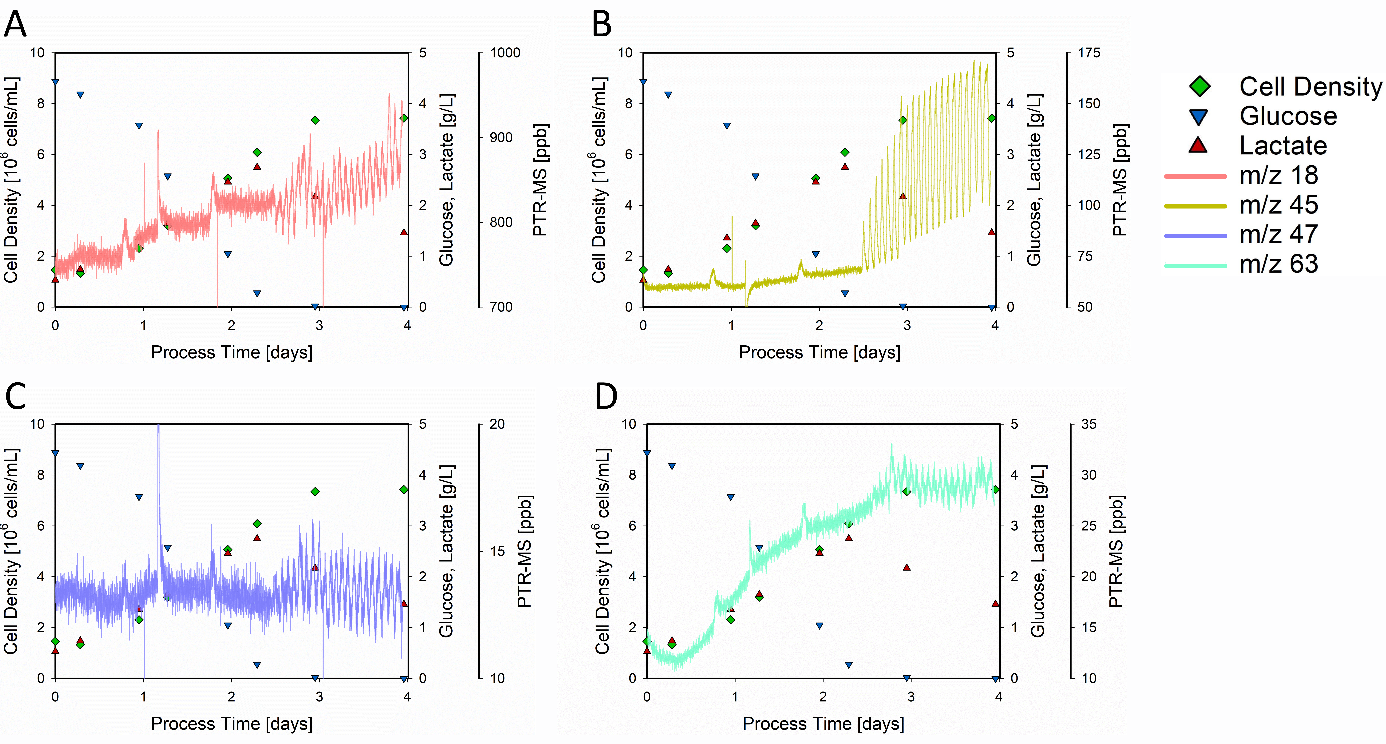
Fig. S3.** Comparison of the usability of different m/z signals as metabolic markers in HEK 293 cultivations. For one non-transfected cultivation, the cell density (green diamonds), glucose (blue triangles), and lactate (red triangles) concentrations are displayed along with the four VOC candidates. The VOCs are represented by solid lines: m/z 18 (A, red), m/z 45 (B, dark yellow), m/z47 (C, blue), and m/z 63 (D, turquoise).

From the start to the finish of the cultivation, the measured PTR-MS values for ammonia (Fig. S3A, m/z 18) were permanently increasing (approximately 30 %) and overall noisy. In contrast, acetaldehyde (Fig. S3B, m/z 45) proved to be almost noise-free with a constant trend until the glucose in the cultivation is depleted (~2.5 days). As soon as glucose is depleted, this PTR-MS signal displayed high fluctuations and a 185 % increase of the detected concentration (from ~60 ppb to ~170 ppb), indicating a switch in the metabolism. Ethanol (Fig. S3C, m/z 47) displayed a constant but noisy trend over the complete process time with low ppb values. Also, ethanethiol (Fig. S3D, m/z 63) was only detectable in small but slowly rising amounts.

Even though all m/z signals reacted to glucose depletion, e.g., increased fluctuations, m/z 45 proved to be the best choice due to the explicit characteristic of suddenly high fluctuating values in contrast to an almost steady trend before. Therefore, m/z 45 was chosen as the metabolic marker for glucose depletion and further utilized in the main manuscript.
